# Movement Control and Long-Latency Reflexes Are Reproducible Measures of Shoulder Neuromuscular Control

**DOI:** 10.3390/jfmk11020150

**Published:** 2026-04-08

**Authors:** Chishan Shiao, Olga Dubey, Michael A. Petrie, Clayton L. Rosinski, Matthew A. Howard, Richard K. Shields

**Affiliations:** 1 Department of Physical Therapy and Rehabilitation Science, Carver College of Medicine, University of Iowa, 1-252 Medical Education Building, Iowa City, IA 52242, USA; 2 Department of Neurosurgery, Carver College of Medicine, University of Iowa, Iowa City, IA 52242, USA

**Keywords:** unexpected perturbation, long-latency reflex, reliability, visual motor task, upper extremity

## Abstract

**Background**: Human physiology-based biomarkers, such as transcortical long-latency reflexes (LLRs) and movement control performance, are measurements used to evaluate human performance. We developed a method to assess human performance variables using a custom-designed visuomotor control device with the capability to examine performance accuracy and neurophysiological responses to unexpected perturbations. We assessed the internal consistency and reproducibility of this device during a shoulder tracking task including the performance accuracy and the transcortical long-latency reflexes during unexpected perturbations. **Methods**: 86 healthy young adults (49 females, right-handed, mean age 25.8 ± 9.5 years) were assessed for internal consistency across varying resistance and velocity conditions. We next determined test–retest reliability among 31 participants (17 females, right-handed, mean age 24.13 ± 3.8 years). We then determined the predictability of the LLRs and performance error during perturbations using the receiver operating characteristic analysis (ROC). **Results**: Our results supported excellent internal consistency, fair-to-good test–retest reliability for task performance accuracy, and fair-to-good transcortical LLR responses to perturbations (McDonald’s omega > 0.9; intraclass correlation coefficients (ICCs, 0.63–0.82)). Tracking accuracy, changes in movement velocity, and infraspinatus LLRs were effective predictors of perturbation conditions (receiver operating characteristics: AUC 0.72–0.90). **Conclusions**: These findings support that performance-based biomarkers have moderate-to-good reliability and neurophysiology-based biomarkers have fair-to-good reliability when assessing human shoulder performance among healthy adults. Studies are currently underway to determine if these measures are reproducible across other joint movements and among people with musculoskeletal and central nervous system injury.

## 1. Introduction

The development of biomarkers grounded in human movement and physiology is important to document changes in human function and performance. These biomarkers can serve as objective measures to track changes in human physiological responses to rehabilitation interventions, surgical treatments, drug trials, or natural recovery after musculoskeletal injuries and from central nervous system (CNS) injuries [1,2,3]. By establishing reliable biomarkers as indicators of change, researchers and clinicians can develop trials to measure functional change among people with and without movement control compromise.

CNS diseases and injury can impair neuromuscular control for visuomotor tasks, altering both performance metrics and physiological responses. Movement accuracy and velocity matching skills are commonly used as performance indicators to assess visuomotor control. As for muscle reflexive responses, long-latency reflexes (LLR) play a key role in feedback control, as they are task dependent and transcortical [4,5,6]. Studies have discovered that supraspinal contributions to LLRs include the primary motor cortex and cerebellum, providing knowledge of limb dynamics and modulating neural pathways to adjust the LLR sensitivity [5,7,8,9,10]. Due to LLR transcortical characteristics, changes in CNS function alter the amplitude of the LLRs as measured by muscle electromyographic activity (EMG). Abnormal LLRs are observed among people with stroke, Parkinsonian syndromes, Huntington disease, muscular dystrophy, and multiple sclerosis [11,12,13,14,15,16,17]. Abnormalities of LLRs may be promising neurophysiology-based indicators of CNS function if they can be reproducibly measured. Developing reliable functional measurements that are congruent with the underlying movement control neurophysiology (LLR) is important for clinical efficacy trials.

To assess human performance and the LLR, a visuomotor task assessment device was developed, where upper extremity movement performance was assessed in response to movement-based unexpected perturbations. When the moving limb is perturbed, by cutting the resistance to a braking mechanism, a transcortical LLR is triggered consisting of an agonist inhibition and antagonist excitation to generate a torque to offset the error and bring the limb to a stop [5]. During LLR assessment, participants are instructed to track a target as accurately as possible and at various speeds and resistances and are provided with feedback of their performance after the test. At any time, the resistance can drop to a pre-programmed level triggering an unexpected perturbation and an LLR, an event that is common in human movement when we lift an object that is heavier than we expected, causing an impending stretch and need for feedback control. Because the user is focused on obtaining a high score on the tracking task, the central set may be controlled during subsequent perturbations, rendering a method that may be highly reproducible for delivering a perturbation in cross-sectional and longitudinal studies of human performance.

In this study, we aspired to establish the scientific rigor of several of the measurements generated during this novel visuomotor assessment to understand reproducibility and interpretability of research findings in the field, in alignment with NIH guidelines [18]. Specifically, we sought to determine the internal consistency of the device for a battery of movement tasks involving varied resistance and velocity settings, examine the test–retest reliability of the motor performance battery and the associated neurophysiological responses (LLRs), and assess the discriminative utility of each biomarker to determine the probability when a true perturbation occurs at the shoulder. We expect that this visuomotor movement test battery will show good to excellent internal consistency, fair-to-good reliability, and fair-to-good predictive capacity of a perturbation and warrant future studies among other joints in people with and without musculoskeletal and CNS pathology.

## 2. Materials and Methods

### 2.1. Participants

Eighty-six young healthy participants (49 females, all right-handed, 25.8 ± 9.5 years old) were recruited. Some of the participants (31 participants, 17 females, all right-handed, 24.13 ± 3.8 years old) were asked to come twice to assess longitudinal test–retest reliability of the visuomotor assessment. All participants had no visual impairment and had not experienced any pain during activity or at rest, surgical or non-operative treatment, or a neurological disorder affecting movement and range of motion of left shoulder. Caffeine, alcohol, and excessive work-out of the left shoulder were prohibited prior to testing and confirmed by all participants via a questionnaire prior to enrollment in the study. This study was approved by the Review Board of the University of Iowa (No. 201407730), and written informed consent was obtained after explanation of the study. All experiments were in accordance with the Declaration of Helsinki.

### 2.2. Experimental Design

All participants performed a baseline test of the visuomotor assessment that was repeated one week later. In both tests, participants were instructed to perform a shoulder tracking task with wireless electromyogram (EMG) electrodes (Delsys Trigno, Natick, MA, USA) placed on the left infraspinatus, pectoralis clavicular, and pectoralis sternal muscles according to SENIAM guidelines [19] to measure muscle activation during shoulder external and internal rotation movements. We selected the shoulder because it is often considered the most difficult area to capture repeatable EMG using surface electrodes. We used the infraspinatus because it is one of the rotator cuff muscles, which is key to shoulder joint stability, often has pathology, and is a primary external rotator of the shoulder. In this task, the infraspinatus would be the agonist and expected to trigger an inhibition of the LLR during perturbation into external rotation. We also measured the two heads of the pectoralis major muscle as the pectoralis sternal major is a primary shoulder internal rotator while the pectoralis clavicular major is both a flexor and an internal rotator of the shoulder. In this task, the two heads of the pectoralis muscle would be the antagonists and expected to trigger an excitation of the LLR during perturbation, with the sternal component being aligned biomechanically to be the most sensitive to a stretch.

Maximum volitional isometric contractions (MVICs) of shoulder external and internal rotation were assessed before the tracking tasks were performed and used to normalize all EMG recordings each day. Reproducibility of the MVIC force showed high correlations (r = 0.96–0.99, *p* < 0.001). The EMG during the MVICs also showed high reliability coefficients (r = 0.90–0.98, *p* < 0.001). Because of known variance in day-to-day skin impedance, all EMGs were normalized to each day’s MVIC EMG.

### 2.3. Instrumentation

**Figure 1:**
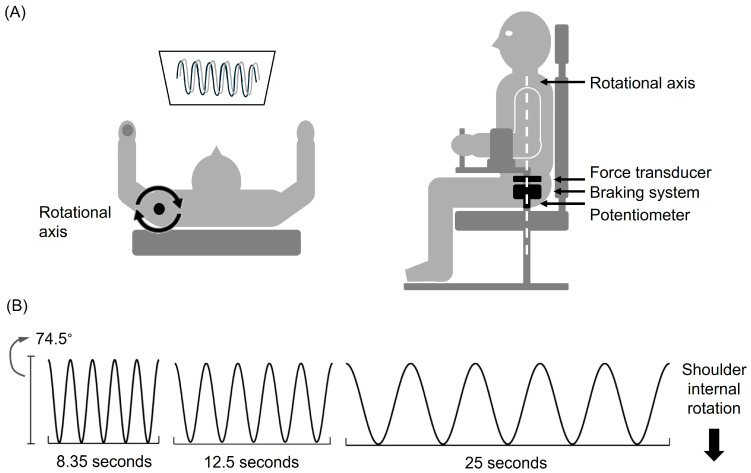
Illustration of (**A**) the Neuromuscular Therapeutic Training System and (**B**) the shoulder tracking task. (**A**) The left panel shows a top view of the system, and the right panel shows a side view. The left handle of the chair is movable along the rotational axis of the shoulder joint for participants to perform shoulder rotation. Target waveform (the black trace) and immediate visual feedback of actual shoulder position (the gray trace) were displayed on a screen in front of the participant. Force transducer, braking system, and potentiometer were attached under the left handle along the rotational axis. (**B**) The shoulder tracking task consists of five cycles of the sinusoidal target waveforms with slow, medium, and fast velocity levels.

The Neuromuscular Therapeutic Training System (NTTS) was custom designed and built in our laboratory (Iowa City, IA, USA). The design enabled us to deliver unexpected perturbations during the shoulder tracking task (Figure 1). This NTTS device consists of an adjustable chair, a force transducer, an electromechanical braking system, and a potentiometer. The left handle of the chair is movable along the rotational axis of the shoulder joint, so participants can rotate the handle horizontally in a position of shoulder flexion at 0 degrees, shoulder abduction at 40 degrees, and elbow flexion at 90 degrees. During the shoulder tracking task, the left forearm was strapped on the handle to ensure the handle moves along with shoulder movements.

The custom-built NTTS is based on a similar device in our lab for the lower extremity (13). The NTTS is designed to measure and control rotational movements of the left shoulder, specifically between 37.52° external and 37.52° internal rotation. The device features a mechanism to record angular displacement, measured by a potentiometer coincident with the axis of rotation of the shoulder. Resistance is provided by an electromagnetic braking system, controlled via a microcomputer with a digital-to-analog interface under customized software control. The brake applies constant resistance proportional to each participant’s maximum voluntary isometric contraction (MVIC) of the left shoulder external rotation. Each participant was seated in the chair with their left arm and forearm secured with fitted cuffs, while the left hand gripped a vertical handle to ensure stability. The left shoulder was strapped to restrict vertical translation of the joint, and the right arm was resting on an armrest to maintain a stable posture. Calibration of the NTTS was completed by attaching an external torque motor (Biodex Medical Systems, Shirley, NY, USA) to the shaft and delivering varying resistance levels to the brake across the angular velocities evaluated in this study. The linearity, repeatability, and hysteresis errors were all within 0.5% of full scale for the brake range. The brake achieved zero resistance within 10–15 milliseconds of release, and this delay was taken into consideration when establishing the EMG time bins for LLR analysis. The force transducer and potentiometer were also calibrated independently and showed excellent linearity, hysteresis, and repeatability levels that were all less than 0.1% of full scale. The braking system was used to provide resistance, and, at a precise time, an unexpected perturbation was delivered by releasing the resistance of the brake randomly during the external rotation phase, lasting 500 ms, 250 ms, and 200 ms in slow, medium, and high velocity trials. After the perturbation was delivered, the resistance returned to the same pre-perturbation level. The rate of change in force when the brake was released at the time of the perturbation and the rate of change in the user displacement were highly correlated (r = 0.85, *p* < 0.001).

### 2.4. Shoulder Tracking Task

To perform the shoulder tracking task, participants sat in the device and faced a screen at eye level. The screen displayed a target moving in a sinusoidal waveform and leaving a white line along its path. Participants were instructed to track the target as accurately as possible by controlling a red line with shoulder rotation. Starting from the shoulder externally rotated position, participants moved to the internally rotated position and returned to external rotation to complete one cycle. The peak of the wave corresponded with 37.52 degrees of external rotation and the valley of the wave corresponded with 37.52 degrees of internal rotation.

People have various skill levels in human performance. In an effort to make the assessment generalizable to multiple skill levels we developed a test battery with 9 different combinations of resistance and velocity levels. These 9 conditions offered an estimate of the population of movement conditions using this device so that we could assess the generalized reproducibility. The resistance levels were set at 10 (low), 15 (medium), and 20 (high) % of pre-test MVICs of the external rotators. These resistance levels were chosen relative to the external rotation because the infraspinatus is an “agonist” for the associated perturbation as an external rotator. The level of resistance and velocities were chosen based on extensive pilot studies to ensure that the task was not fatiguing and that a given perturbation could trigger an LLR at a safe level. After the brake release, the underlying maximal target angular velocity, internal rotator torque, and joint reaction torque were modeled to be less than 3 rad/s, 25–35 Nm, and 20–30 Nm, respectively. The target movement angular velocities during the movement were 30 (0.2 Hz, slow), 60 (0.4 Hz, medium), and 90 (0.6 Hz, high) degrees/second. The mean angular velocity (degrees/s) after brake release for each of the 9 test conditions in this study were 70.0 (+/−0.76), 69.7 (+/−0.83), and 75.5 (+/−0.82) for the low, medium, and high resistance, respectively, at the low speed (0.2 Hz); 85.3 (+/−2.2), 88.1 (+/−1.0), and 91.3 (+/−2.1) at the low, medium, and high resistance, respectively, at the medium speed (0.4 Hz); and 106.0 (+/−4.3), 113.3 (+/−4.6), and 116.4 (+/−5.0) at the low, medium, and high resistance, respectively, at the high speed (0.6 Hz). Accordingly, our actual average angular velocities in this study as a result of the perturbation were kept below our modeled estimate of 3 rad/s (180 degrees/s). Each condition contained 5 cycles of the sinusoidal waveform. Unexpected perturbations were delivered randomly in one of the five cycles of each condition, except for the first cycle. In baseline and post-test, participants had to perform all 9 conditions of the task without knowing the task order. A practice trial was given before the first trial in the baseline test for participants to understand the task better, and a 30 s rest was provided between each condition.

### 2.5. Measurements

**Figure 2:**
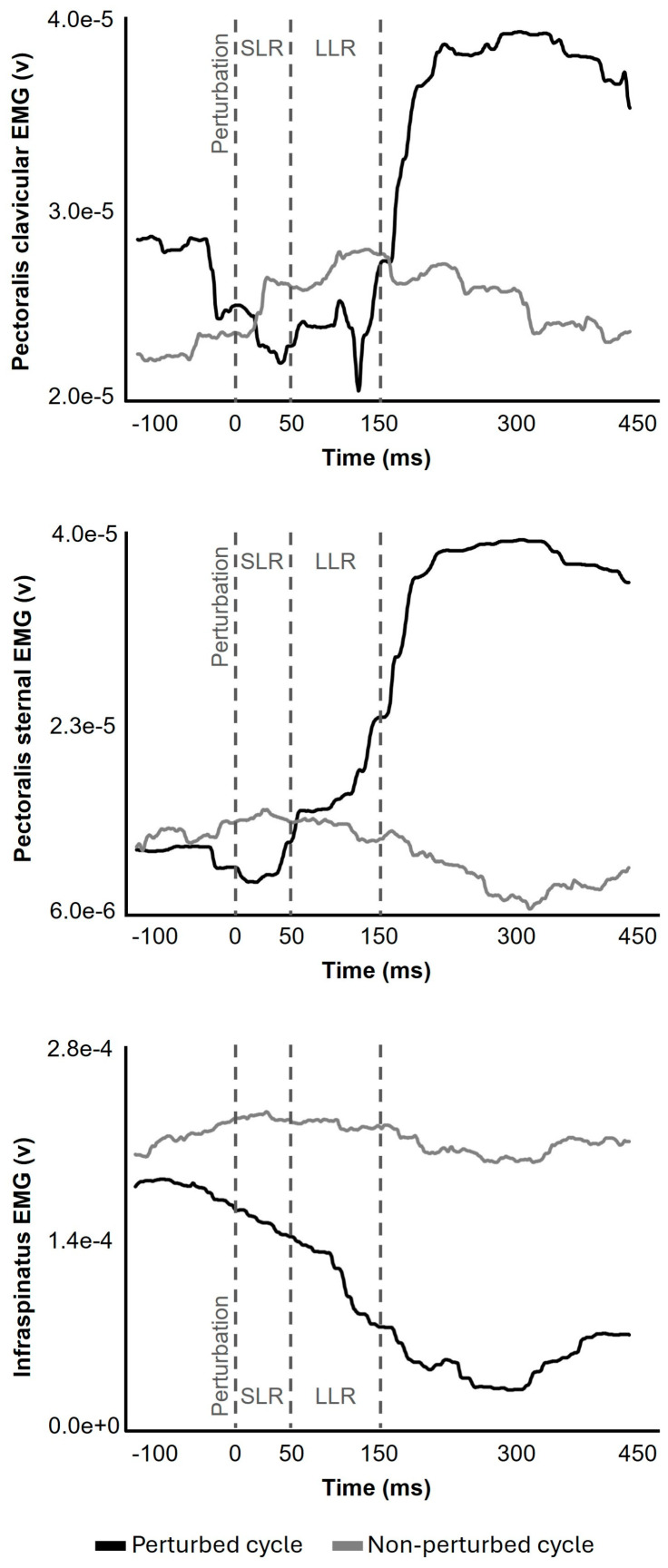
An example of root mean square values of the EMG signal recorded from the pectoralis clavicular, pectoralis sternal, and infraspinatus muscles during a perturbed cycle (black) and non-perturbed cycle (gray). SLR: short-latency reflex; LLR: long-latency reflex.

We measured coherence, mean absolute error, peak absolute error, and peak user rate error to assess performance during the shoulder tracking task. Coherence represents the ability to match the velocity of the sinusoidal waveform throughout all 5 cycles of each condition. Coherence values range between 0 and 1, where 0 means the user velocity is completely unmatched with the sine wave velocity, and 1 means a linear match. Absolute error, representing tracking accuracy, was calculated as the absolute value of the difference between the target and the shoulder displacement at each time point. Mean and peak absolute error are the average and highest value of absolute error obtained from each condition, respectively. Peak user rate is the peak value of the instantaneous user signal’s slope within 50 ms after perturbation (0–50 ms time bin), representing the change in shoulder movement velocity caused by perturbation. EMG signals were collected with a 20–450 Hz bandwidth, common mode rejection ratio > 80 dB, and 16-bit resolution, and were sampled at 2000 Hz. Using a custom DIAdem script (National Instruments, Austin, TX, USA, v.2012) for EMG signal analysis, the root mean square of the EMG signals was calculated and was reported as a percentage of the peak MVIC EMG in the LLR (50–150 ms) time bin (Figure 2). Perturbed cycles of each condition are the cycle when perturbation occurred, and non-perturbed cycles are the three cycles without perturbation, excluding the first cycle.

### 2.6. Statistical Analyses

We used McDonald’s omega to assess internal consistency of measurements across 9 conditions of shoulder tracking task to investigate whether LLR can be measured in all conditions using the NTTS device [20,21]. Data obtained from all cycles of each condition was used in performance measures, and data obtained in perturbed cycles of each condition were used for LLR amplitude. Cronbach’s alpha was also reported as recommended for comparative analysis [20,21,22]. To assess test–retest reliability of measurements across baseline and post-test, we determined the absolute reliability using standard error of measurement (SEM) and intraclass correlation coefficients (ICCs). SEM is calculated as SEM = SD√(1 − ICC), with smaller value indicating higher reliability [23,24,25]. Intraclass correlation coefficient (ICC) estimates and their 95% confidence intervals (CI) were calculated based on a single-rating, absolute-agreement, two-way mixed-effects model that was introduced in Koo and Li [26,27,28]. ICCs with values < 0.5, 0.5–0.75, 0.75–0.9, and >0.9 indicate poor, moderate, good, and excellent reliability, respectively [23,24,25,26]. For performance measures and LLR amplitude, we used the average values of the 9 conditions with perturbed and non-perturbed cycles to assess test–retest reliability, but also calculated ICCs for each of the 9 conditions. Prior to calculating the ICCs for coherence, we transformed the data using a Fisher z-transformation because correlational data are not normally distributed. To understand each measurement’s capability to detect true perturbations for when perturbation was delivered, the receiver operating characteristic (ROC) curve and the area under the curve (AUC) were calculated [29,30]. AUC values > 0.7 are generally considered acceptable [29]. We used the combined data of the perturbed and non-perturbed cycle of each test to determine the perturbation-predictive ability of performance measures and LLR amplitude, but for comparative purposes performed the analysis across each velocity/resistance condition. Statistical analyses were conducted using R Statistical Software (v4.4.3; R Core Team 2024).

## 3. Results

### 3.1. Internal Consistency

Performance measures such as coherence, mean and peak absolute error yielded McDonald’s omega and Cronbach’s alpha values ranging from 0.85 to 0.96 (Table 1). Similarly, the LLR amplitude of the pectoralis clavicular, pectoralis sternal, and infraspinatus during the perturbed cycle showed omega and alpha values between 0.89 and 0.96. These results support a high degree of internal consistency across all conditions with varying levels of resistance and velocity for both performance and reflexive response.

### 3.2. Test–Retest Reliability

Relative test–retest reliability of the performance-related variables showed that mean and peak absolute errors, coherence, and peak user rate demonstrated moderate-to-good test–retest reliability (Table 2). Notably, the 95%CI for the mean absolute error was broader than that of the peak absolute error, with a lower bound of 0.27, suggesting that the peak absolute error estimated more precisely than the mean absolute error. The LLR amplitude of the pectoralis sternal and infraspinatus presented good test–retest reliability, showing greater relative test–retest reliability compared to the moderate test–retest reliability of the pectoralis clavicular.

The absolute test–retest reliability results reported the SEM of each measure, presenting the changes that should be considered as real changes rather than systemic measurement errors (Table 2). Among performance measures, the peak user rate showed a slightly higher SEM, indicating that a change in peak user rate greater than 3 degrees/second is needed to be considered as an actual difference. The infraspinatus LLR amplitude showed the highest SEM among the three muscles. We also calculated the minimal detectable change (MDC) at the 95%CI of each indicator to understand how much change is needed to show an effect. According to the equation of MDC at 95%CI = SEM × √2 × 1.96 [31,32], the MDCs of performance measures from the smallest to the largest are 0.542 for coherence, 3.093 degrees for the mean absolute error, 5.721 degrees for the peak absolute error, and 9.298 degrees/second for the peak user rate. As for the LLR amplitude, the MDCs from smallest to largest are 6.70% MVIC for the pectoralis sternal, 8.93% MVIC for the pectoralis clavicular, and 13.7% MVIC for the infraspinatus. When we examined the ICCs for each of the nine conditions we show similar results as the entire test battery with moderate-to-good ICCs for the mean absolute error (range: 0.72–0.64), peak absolute error (range: 0.78–0.71), peak user rate (range: 0.67–0.55), infraspinatus (range: 0.63–0.59), pectoralis sternal (range: 0.59–0.55), and pectoralis clavicular (range: 0.55–0.46). (See Supplement Tables S1 and S2).

### 3.3. Probability of Detecting True Perturbation

**Figure 3:**
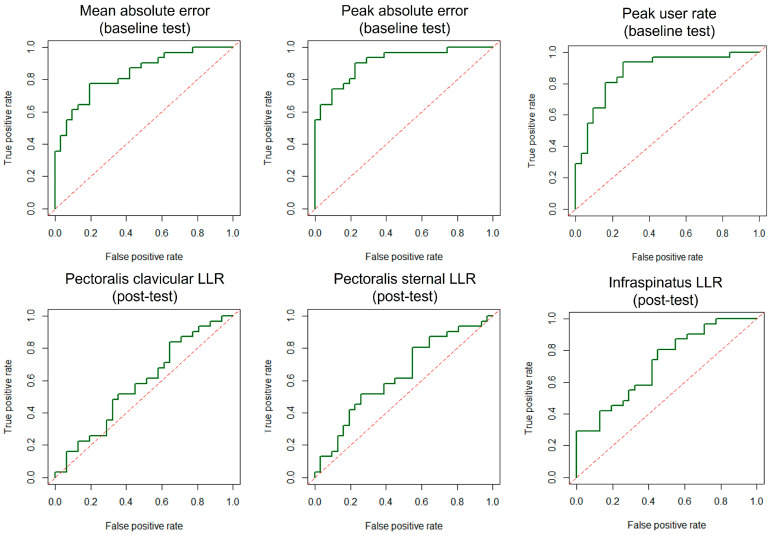
ROC curves of peak absolute error, peak user rate, and LLR amplitude of pectoralis clavicular and pectoralis sternal. LLR: long-latency reflex.

The ROC curve and AUC were established for all measurements to quantify the predictive capability for perturbation. The performance measures showed higher AUC values than those derived from the LLR amplitude (Table 3 and Figure 3), indicating performance measures are more responsive to perturbation than the LLR amplitude. In the baseline test, the peak absolute error demonstrated good predictive ability for perturbation occurrence, whereas absolute error and peak user rate indicated fair capability. The mean absolute error also showed the lowest AUC values in the post-test; however, the peak user rate in post-test exhibited a higher AUC than the peak absolute error in post-test, though both metrics still fell within the fair prediction range. The LLR amplitude of the pectoralis clavicular and sternal displayed failed and poor predictive capability in baseline and post-test, respectively. The infraspinatus LLR amplitude presented poor and fair predictive capability in baseline test and post-test, respectively. Condition-specific AUC analyses showed similar results to general AUC analysis, with fail-to-poor predictive capability of the pectoralis clavicular LLR amplitude in baseline (AUC 0.56–0.60) and post-tests (AUC 0.56–0.68), fail-to-poor capability of the pectoralis sternal LLR amplitude in baseline (AUC 0.53–0.65) and post-tests (AUC 0.56–0.68), and poor-to-fair capability of infraspinatus amplitude in baseline (AUC 0.63–0.76) and post-tests (AUC 0.66–0.77). There was a trend for AUCs to be slightly higher for the infraspinatus during conditions with higher resistance.

## 4. Discussion

The primary findings support that this battery of visuomotor tasks demonstrates (1) excellent internal consistency across performance measures and LLRs, (2) good-to-moderate test–retest reliability in the performance measures, (3) moderate-to-good test–retest reliability in the trans-cortical LLR amplitude, and (4) good discriminative utility of the peak absolute error, mean absolute error, and peak user rate as predictors of perturbations; and (5) poor-to-fair discriminative utility of LLRs as predictors of perturbations. Taken together, the performance measures during the perturbation were more invariant than the underlying neural control strategies (LLRs) that were deployed to try to control the magnitude of the perturbation.

Compared to all performance measures assessed, the peak absolute error was the most reliable performance indicator of visuomotor control. Using the peak absolute error, the assessment device demonstrated excellent internal consistency and good relative test–retest reliability. Moreover, the peak absolute error demonstrated good ability to predict whether a perturbation occurs, suggesting it is responsive to perturbation occurrence. Although the peak and mean absolute error both represent tracking accuracy, the peak absolute error had a higher ICC and AUC compared to the mean absolute error. A higher ICC indicates greater between-group variation in the peak absolute error, which may be attributed to the inherent sensitivity of peak values to transient deviations, as these errors are not removed by averaging. The narrower 95%CI of the ICC in the peak absolute error also supports that the peak absolute error estimated the ICC more precisely than the mean absolute error. A higher AUC may also be attributed to the greater sensitivity of the peak absolute error to perturbations compared to the mean absolute error. The greater between-group variation in the peak absolute error may result from the difficulty of tracking the target line accurately throughout a whole condition, compared to simply matching the velocity of the sinusoidal waveform. Together, these findings highlight the peak absolute error as a reliable indicator for detecting perturbations.

The long-latency reflex amplitudes of the pectoralis sternal and infraspinatus muscles showed better overall results with high internal consistency (Cronbach’s Alpha), moderate-to-good test–retest reliability (ICCs), but poor-to-fair discriminate utility in perturbation prediction. Physiologically, the infraspinatus was the agonist, requiring a rapid inhibition within the LLR timeline (50–150 milliseconds) to stop the limb before volitional reaction time can have an effect. The notion that this rapidly occurring LLR inhibition can be measured with good reliability and fair predictability of a perturbation is an important finding given the multiple neural control strategies (redundancy) that the CNS has to bring a limb to an abrupt stop. Varying antagonist muscle excitation in combination with varying degrees of agonist inhibition offers a range of strategies that may be unique to better performance across individuals. While speculative, it may be that future studies, via cluster analysis, will be able to identify the CNS synergistic strategies that are most effective in offsetting the mechanical changes induced during an unexpected event.

Importantly, in this study, the muscle with the least biomechanical contribution to the movement (pectoralis clavicular) was the poorest predictor of a perturbation and showed lower reproducibility. The pectoralis clavicular and pectoralis sternal generate shoulder internal rotation when contracting together. However, when acting independently, the pectoralis clavicular flexes the forearm, and the pectoralis sternal extends the forearm [33]. Due to this difference, the pectoralis clavicular may activate less efficiently during this task. As a result, there was less between-group variation in the LLR amplitude of the pectoralis clavicular compared to that of the pectoralis sternal.

In this study, we also provided minimal detectable change (MDC) for performance and LLR measurements. Investigation of MDC enables examiners to know the minimum differences in indicators to show meaningful changes and reveals an evaluative instrument’s responsiveness, which is the likelihood that the instrument can detect a change outside of systematic error [31,32,34,35]. According to our results, the LLR should show a difference larger than 6.701% MVIC to support a meaningful change in the LLR amplitude of the pectoralis sternal, and differences larger than 8.932% and 13.74% MVIC are needed for the LLR amplitude of the pectoralis clavicular and infraspinatus, respectively. The magnitude of MDCs was not surprising for the infraspinatus as there are several other external rotators of the shoulder that may be recruited as part of the movement strategy during a perturbation, and which we were not able to record with surface electrodes. Notably, these MDCs are determined from a healthy population, so MDCs of performance measures and LLR amplitudes of people with impairment are not known. Because CNS impairment often reduces degrees of freedom for movement strategies, it may be that they become more invariant among those with impairment and more reproducible as compared to healthy. Understanding the psychometrics of movement control parameters is needed for all perturbation studies and is congruent with the new NIH guidelines.

However, few studies have examined the reliability of movement performance and physiological variables after perturbation of the shoulder in humans. Recently, Heinke and colleagues [36] studied the reliability of the short-latency reflex amplitude of the pectoralis under three levels of pre-load before stretch (0%, 10%, and 25% MVIC) and showed moderate-to-good reliability of pectoral muscle reflex responses. Perturbation was delivered by a stretch induced by a torque motor starting in a static position with various pre-loads before stretching. Our work nicely complements their work based on our similar levels of reliability of reflex responses, but adds a novel dimension in that our perturbation was superimposed during a human movement control task at various levels of resistance and velocities and for which the performance could be measured. The central set of a person who has received perturbation from an external torque motor likely has a different experience than a person who is fooled during a goal-oriented movement task [5]. Both types of studies are important and need rigorous measures to better understand the strategies that are effective in responding to unexpected events. One physiological strategy that may be used by both experimental approaches is to optimize the modulation of the alpha motor neurons and the gamma motor neurons as they regulate the synergists and muscle spindle sensitivity during a stretch [37,38,39,40]. It is noteworthy that the peak angular velocities of the perturbed limb in our studies were ~75, 91, and 116 degrees/s for the three tracking task speeds, respectively (0.2, 0.4, and 0.6 Hz), below our estimate based on our pilot modeling peak velocity (180 degrees/s). Our angular velocities were less than 150 degrees/s, which is congruent with a recent perturbation study that used a torque motor and background isometric contraction [36].

Overall, the test battery of movements from this study appears to be generalizable given the range of task performances studied across a large sample size, and the fact that we constrained the participants to use their non-dominant limb. By using the non-dominant limb, we attempted to make our group more homogenous as the task would be considered a more novel task, but this is also a limitation in that we do not know how the dominant side would react. We used the aggregate of the full battery of tests to determine our test–retest sample size for reliability precision (power = 0.86). When analyzed for each individual velocity-resistance setting, the reliability and AUC levels were internally consistent with the overall aggregate findings, but we observed a non-significant trend for lower ICC values with faster velocities. Our findings generally support the idea that people who can only move at a lower velocity may be able to generate more reliable perturbations and associated performance outcomes. We did not observe a similar trend for improved ICCs with increased velocity with the LLR analysis. Importantly, given our sample size, our power fell below the 0.80 level when examining test–retest reliability for some velocity-resistance conditions. Future studies are needed, with a larger sample size, to determine reliability for each specific velocity-resistance condition.

There are several limitations in this study including that these findings pertain only to the non-dominant shoulder joint and that only a limited number of muscles were studied that rotate the joint in healthy adults. Thus, a CNS strategy involving the recruitment of a separate internal or external rotator that was not recorded may explain why some participants could show a lower reproducibility. The shoulder joint itself, being a highly unstable joint with limited access to all synergists, is challenging when compared to a more stable joint like the elbow. Importantly, we were slightly underpowered in test–retest reliability precision for each velocity-resistance condition. We speculate that other sources of variation that warrant future assessment are whether the CNS strategy changes were associated with a change in performance (learning). A final limitation of this work is that we do not provide comprehensive modeling of the muscle and mechanical properties of this novel system, an area that is needed in the future. Additional studies are underway to study other joints and patients with various musculoskeletal and neuromuscular impairments to better understand human responses to unexpected events [13].

## 5. Conclusions

Physiologically based biomarkers for transcortical long-latency reflexes can be reliably assessed using a novel visuomotor task assessment device. The findings support good reliability for performance measures and fair-to-good reliability when assessing physiological LLR of shoulder muscles. The peak values of tracking accuracy and the LLR amplitude of the pectoralis sternal and infraspinatus were found to be more reliable biomarkers than the clavicular pectoralis. Future studies in people with various musculoskeletal and neurological diseases are warranted to help uncover the underlying strategies used to adapt the neuromuscular control system.

## Figures and Tables

**Table 1:** Internal consistency of performance measures and LLR in shoulder tracking task.

	McDonald’s Omega	Cronbach’s Alpha
***Performance***		
Coherence	0.93	0.85
Mean absolute error	0.96	0.91
Peak absolute error	0.90	0.86
***LLR in perturbed cycle***		
Pectoralis clavicular	0.92	0.89
Pectoralis sternal	0.93	0.90
Infraspinatus	0.96	0.93

Abbreviations: LLR, long-latency reflex.

**Table 2:** Test–retest reliability of performance measures and LLRs in shoulder tracking tasks.

	ICC	SEM
***Performance***		
Coherence	0.63 (0.46–0.71)	0.195
Mean absolute error	0.75 (0.27–0.80)	1.116
Peak absolute error	0.82 (0.78–0.85)	2.064
Peak user rate	0.77 (0.73–0.80)	3.355
***LLR***		
Pectoralis clavicular	0.57 (0.51–0.63)	3.784
Pectoralis sternal	0.82 (0.78–0.84)	1.692
Infraspinatus	0.77 (0.74–0.81)	8.159

ICC and 95% confidence interval is presented. SEM is presented in degree for absolute errors, degree/second for peak user rate, and % MVIC for LLR amplitude. ICC values: <0.5, poor; 0.5–0.75, moderate; 0.75–0.9, good; >0.9, excellent reliability. Abbreviations: ICC, intra-class correlation; LLR, long-latency reflex; MVIC, maximum volitional isometric contraction; SEM, standard error of measurement.

**Table 3:** Perturbation prediction of performance measures and LLRs in shoulder tracking task.

	AUC	95%CI
***Performance***		
* Baseline test*		
Mean absolute error	0.838	0.740–0.935
Peak absolute error	0.908	0.837–0.980
Peak user rate	0.877	0.789–0.966
* Post-test*		
Mean absolute error	0.783	0.658–0.908
Peak absolute error	0.850	0.747–0.953
Peak user rate	0.867	0.772–0.962
***LLR***		
* Baseline test*		
Pectoralis clavicular	0.578	0.433–0.722
Pectoralis sternal	0.568	0.423–0.713
Infraspinatus	0.674	0.539–0.809
* Post-test*		
Pectoralis clavicular	0.653	0.515–0.792
Pectoralis sternal	0.630	0.489–0.770
Infraspinatus	0.717	0.590–0.844

AUC values: 0.5–0.6, fail; 0.6–0.7, poor; 0.7–0.8, fair; 0.8–0.9, good; ≥0.9, excellent. Abbreviations: AUC, area under the curve; LLR, long-latency reflex.

## Data Availability

The original contributions presented in this study are included in the article/Supplementary Materials. Further inquiries can be directed to the corresponding author.

## Supplementary Materials

The following supporting information can be downloaded at: https://www.mdpi.com/article/10.3390/jfmk11020150/s1, Table S1: Test-retest reliability of performance measures in various shoulder tracking condition. Table S2: Test-retest reliability of LLLs in various shoulder tracking conditions.

## Abbreviations

The following abbreviations are used in this manuscript:

AUC	Area under the curve
CNS	Central nervous system
EMG	Electromyography
ICC	Intraclass correlation coefficients
LLR	Long-latency reflex
MVIC	Maximum volitional isometric contraction
MDC	Minimal detectable change
NTTS	Neuromuscular therapeutic training system
ROC	Receiver operating characteristic analysis
SLR	Short-latency reflex
SEM	Standard error of measurement
