# Supplementary material for: Movement Control and Long-Latency Reflexes Are Reproducible Measures of Shoulder Neuromuscular Control
Source: J Funct Morphol Kinesiol. 2026 Apr 8;11(2):150. doi: 10.3390/jfmk11020150 (PMC13108043; doi:10.3390/jfmk11020150)
Supplement: Supplementary file 1 [file jfmk-11-00150-s001.zip › jfmk-4136426-supplementary.pdf]

Supplement Table S1 and Table S2

.

Table S1. Test-retest reliability of performance measures in conditions of shoulder tracking tasks.

	Condition	ICC
Performance		
Mean absolute error	1	0.72 (0.67-0.76)
	2	0.71 (0.66-0.75)
	3	0.70 (0.65-0.74)
	4	0.71 (0.66-0.75)
	5	0.68 (0.63-0.73)
	6	0.65 (0.59-0.70)
	7	0.64 (0.58-0.69)
	8	0.65 (0.59-0.70)
	9	0.66 (0.61-0.71)
Peak absolute error	1	0.78 (0.75-0.81)
	2	0.77 (0.74-0.80)
	3	0.76 (0.73-0.80)
	4	0.77 (0.73-0.80)
	5	0.74 (0.70-0.78)
	6	0.72 (0.68-0.76)
	7	0.71 (0.67-0.75)
	8	0.72 (0.68-0.76)
	9	0.74 (0.70-0.77)
Peak user rate	1	0.67 (0.63-0.71)
	2	0.66 (0.62-0.71)
	3	0.67 (0.63-0.72)
	4	0.67 (0.63-0.71)
	5	0.58 (0.53-0.64)
	6	0.57 (0.52-0.63)
	7	0.55 (0.50-0.61)
	8	0.57 (0.52-0.63)
	9	0.57 (0.52-0.62)

ICC is presented by ICC coefficient and 95% confidence interval. ICC values: < 0.5, poor; 0.5-0.75, moderate; 0.75-0.9, good; > 0.9, excellent reliability. Abbreviations: ICC, intra-class correlation.

Table S2. Test-retest reliability of LLR in conditions of shoulder tracking tasks.

	Condition	ICC
LLR		
Pectoralis clavicular	1	0.54 (0.51-0.58)
	2	0.48 (0.45-0.52)
	3	0.47 (0.44-0.51)
	4	0.46 (0.43-0.50)
	5	0.49 (0.45-0.52)
	6	0.50 (0.47-0.54)
	7	0.52 (0.49-0.55)
	8	0.55 (0.52-0.58)
	9	0.55 (0.52-0.59)
Pectoralis sternal	1	0.56 (0.53-0.59)
	2	0.55 (0.52-0.58)
	3	0.55 (0.52-0.58)
	4	0.56 (0.53-0.60)
	5	0.57 (0.54-0.61)
	6	0.59 (0.56-0.63)
	7	0.58 (0.55-0.61)
	8	0.58 (0.55-0.61)
	9	0.59 (0.55-0.62)
Infraspinatus	1	0.63 (0.60-0.67)
	2	0.61 (0.58-0.65)
	3	0.60 (0.57-0.64)
	4	0.60 (0.57-0.63)
	5	0.59 (0.56-0.63)
	6	0.60 (0.56-0.63)
	7	0.59 (0.56-0.63)
	8	0.61 (0.58-0.64)
	9	0.62 (0.59-0.65)

ICC is presented by ICC coefficient and 95% confidence interval. ICC values: < 0.5, poor; 0.5-0.75, moderate; 0.75-0.9, good; > 0.9, excellent reliability. Abbreviations: ICC, intra-class correlation; LLR, long-latency reflex.
